# Nonthermal Concentration of Red Beetroot Juice by Reverse Osmosis and Forward Osmosis

**DOI:** 10.1002/fsn3.72222

**Published:** 2026-09-09

**Authors:** Eugenia Avellaneda, Carmen I. Moraru

**Affiliations:** ^1^ Department of Food Science Cornell University Ithaca New York USA

## Abstract

This work reports on the use of reverse osmosis (RO) and forward osmosis (FO) for the nonthermal concentration of red beetroot juice. Commercial thermally concentrated juice (TCJ) diluted to single strength and freshly extracted, not from concentrate juice (NFC) were processed by RO with a thin film composite membrane, at 2400 kPa transmembrane pressure and 22°C–23°C. FO concentration was conducted with a cellulose triacetate membrane, at 25°C, using potassium citrate as osmotic agent. Water flux declined over time in both RO and FO, with a more pronounced decay for RO, indicative of membrane fouling. RO concentration plateaued at 17.3 °Brix ± 0.4 °Brix for TCJ and at 12.3 °Brix for NFC, while FO reached concentrations of 50.9 °Brix ± 2.9 °Brix and 49.7 °Brix for TCJ and NFC, respectively. No significant changes (*p* > 0.05) in juice color and betalain content were observed for any of the membrane concentrated TCJ samples; small decreases in betacyanin content and color occurred after both RO and FO of NFC A combined RO–FO process maintained product quality but did not present benefits in terms of speed of concentration. Overall, this work indicates that FO is a promising nonthermal method for the concentration of beetroot juice, with significant advantages compared to both thermal concentration and RO.

## Graphical abstract

RO and FO were used for nonthermal concentration of red beetroot juice. Single strength juice was concentrated by RO to 17 °Brix, and by FO to 50 °Brix; color and betalain were preserved by both processes. Overall, FO is a promising method for concentrating beetroot juice.

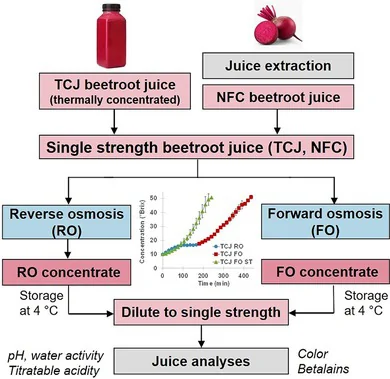

## 1 Introduction

Consumption of red beetroot (*Beta vulgaris L*.) and red beetroot juice has gained increasing interest due to their nutritional, health‐promoting, and disease‐preventing compounds (Mirmiran et al. 2020; Chen et al. 2021). Beetroot is a natural source of bioactive compounds, including betalains, phenols, vitamins, and minerals like manganese, magnesium, potassium, iron, and zinc (Sucu and Turp 2018; Ceclu and Nistor 2020). Betalains are also natural pigments, represented by the red‐purple betacyanins and yellow‐orange betaxanthins (Azeredo 2009). This has led to a growing interest in red beetroot juice as a source of natural colorant (Fernández‐López et al. 2023; Ahmed et al. 2025).

Beet red colorant is available either as juice concentrate or as powder produced by freeze or spray drying of the juice concentrate (Azeredo 2009). Juice concentration is typically done by thermal evaporation, which provides high yields and reaches concentrations of up to 75 °Brix (Drioli and Cassano 2013). However, thermal evaporation also leads to flavor degradation, loss of vitamins and antioxidants, and cooked taste caused by furfural formation (Azeredo 2009; Conidi et al. 2020; Sadowska‐Bartosz and Bartosz 2021). Color is also affected, since betalains undergo degradation when exposed to high temperatures, light, or oxygen (Carle et al. 2004, 2006; Azeredo 2009).

Membrane processing by reverse osmosis (RO) is a promising and increasingly used nonthermal alternative to thermal evaporation for juice concentration. Concentration by RO preserves juice quality, but its performance is limited by membrane fouling under the high transmembrane pressures (TMP) used, as well as concentration polarization. Fouling reduces the water permeability of RO membranes due to accumulation of solids on the surface (predominant) or inside the membrane. RO fouling can be represented by biofouling, organic fouling, inorganic scaling, and colloidal fouling (Jiang et al. 2017). Organic fouling, which is primarily due to polysaccharides, proteins, lipids, and organic acids, is the most common type of fouling during RO of liquid foods and beverages.

Concentration polarization (CP) is caused by the accumulation of rejected solutes close to the membrane surface, which produces a concentration boundary layer that increases the osmotic pressure on the membrane surface, reducing the effective driving force and reducing diffusivity, both of which lower the permeate (water) flux. CP and fouling can also act synergistically, further reducing RO performance. The fouling induced cake layer can promote CP by limiting solute back‐diffusion, while the solute concentration gradient created by CP can drive particle migration toward the membrane surface (Bai et al. 2023).

Fouling and CP have significant consequences, since they decrease the flux, increase RO operational costs by shortening the membrane lifespan and increasing the frequency of chemical cleaning, and limit the achievable product concentration. (Gunathilake et al. 2014) reported a final concentration of 15.1 °Brix and 14.9 °Brix for apple and blueberry juices; (Cassano et al. 2003) reached 13.6 °Brix for carrot juice RO processed at 2500 kPa, and (Nuss et al. 2007) reported 18 °Brix for onion juice at 5500 kPa. Feed pretreatments can be used to mitigate fouling and improve RO performance. Aguiar et al. (2013) reached 28.1 °Brix after RO of enzymatically treated and microfiltered apple juice, while (Echavarría et al. 2012) reached 30.5 °Brix for RO of clarified peach juice; while this is an improvement, the final concentration is still much lower than in thermal evaporation.

Recently, forward osmosis (FO), in which a concentrated osmotic agent (OA) is used to create an osmotic pressure difference between the two sides of the membrane, has emerged as an alternative to RO. FO has typically lower water fluxes than RO at low solids levels of the feed, but can reach product concentration levels comparable to thermal evaporation. (Babu et al. 2006) and (Wang et al. 2022) reached ~60 °Brix for FO of pineapple juice and apple juice, respectively, while (Nayak et al. 2011) reached 52 °Brix and 54.6 °Brix for FO of beetroot and grape juice, respectively.

While fouling and CP are less prominent in FO compared to RO, FO is also affected by both phenomena. In FO, CP can occur as both external concentration polarization (ECP), which refers to the accumulation (concentrative ECP) or reduction (dilutive ECP) of solutes adjacent to the membrane surface, and internal concentration polarization (ICP); both ECP and ICP have a negative effect on the net driving force for water transport (Zhao et al. 2012). Additionally, FO is constrained by osmotic agent (OA) selection and regeneration, and reverse solute flux (RSF). RSF could represent a problem for food applications, where it could be viewed as food contamination, and it may also impact product taste, even at very low levels (Menchik and Moraru I 2019; Beldie et al. 2025). Unfortunately, RSF is often not reported in the literature.

The goals of this work were to compare the efficiency of RO and FO for beetroot juice concentration, individually and in a sequence, to assess the preservation of betalains during these membrane concentration processes, as well as the level of RSF during the FO process. The data presented in this report can inform industry practitioners about the potential of RO and FO as alternatives to thermal evaporation for red beetroot juice concentration and can also represent the starting point for a more in‐depth evaluation of these technologies for juice concentration.

## 2 Materials and Methods

### 2.1 Beetroot Juice

Thermally concentrated beetroot juice (TCJ) of 65 °Brix from Greenwood Associates (Chicago, IL) was used as a stock material for most of the study. Before RO and FO, TCJ was diluted with DI water to single strength, 10 °Brix, the average concentration measured for three commercial beetroot juices (RW Knudsen, ios Organic, and Georgia's Natural).

One batch of freshly extracted, not from concentrate (NFC) beetroot juice, provided by Love Beets (Rochester, NY), was used for a validation study. The juice was obtained from a large amount of beets, which originated from the same source and the same harvest. Frozen beets were thawed, cut into small pieces, heated, and incubated with enzymes until the following day, when juice was extracted with a belt press (Flottweg SE, Vilsbiburg, Germany). The juice was frozen, and immediately before use was thawed and clarified using an Alfa Laval LAPX 404 SGP‐31 separator (Alfa Laval, Pune, India), at 5 L/min feed rate, 5.0% feed solids concentration, 1 L discharge volume, and 90% solids space packed volume. After clarification, 76 kg of juice with 1% suspended solids was collected and stored refrigerated until the following day.

### 2.2 RO Processing

TCJ reconstituted to 10 °Brix was processed using a RO rig (Osmonics, WI) equipped with a spiral‐wound polyamide thin film composite membrane (SW30‐2540, Dupont Water Solutions, MN) of 61 mm outside diameter, 1061 mm length, and 2.69 m^2^ filtration area. The juice was circulated from the feed tank using a centrifugal pump (Tonkaflo SS, 1804—GE, Trevose, PA) and passed through a pre‐filter cartridge. Two booster pumps (Tonkaflo SS 526X 3290‐GE, Trevose, PA) were used to generate a TMP of 2390 ± 90 kPa, the maximum allowed by the equipment. The juice was circulated through a counter‐current heat exchanger to maintain it at 22.9°C ± 0.5°C. The permeate (water) was collected in a container placed on a scale that logged the mass. Temperature was monitored with a thermocouple at the membrane inlet and the flow rates with flow meters on the feed, retentate and permeate sides. Inlet and outlet pressures were measured by pressure gauges, and TMP calculated; the TMP was adjusted with the valve that controlled retentate flow rate. The retentate was recirculated until concentration reached a plateau. The TCJ runs were performed in triplicate. For two of the RO runs, ~74 kg juice were concentrated for 120 min. The third run was extended to 240 min, to confirm the concentration plateau, and resulted in 88.5 kg of product.

For the RO validation run, single strength NFC beetroot juice (~8.4 °Brix) was concentrated as described above, at 22.5°C ± 2.7°C, TMP of 2448 ± 14 kPa, for 240 min.

The final juice concentrate was collected in amber glass bottles and kept refrigerated (4°C ± 1°C) until analyzed the following day.

The RO instant permeate (water) flux (*J*, L/m^2^h) was calculated as follows:

(1) $$J=\frac{\;m_{p}}{A\times t\times \rho }$$

where *m*_*p*_: mass of permeate (kg), *A*: filtration area (m^2^), *t*: time (h), *ρ*: density of water (1 kg/L).

The feed concentration was measured every 15 min with a digital refractometer (Sper Scientific 300,053‐Scottsdale, AZ) and used to calculate the concentration factor (CF):

(2) $$\mathrm{CF}=\frac{{}^{\circ}{\text{Brix}}_{t}}{{}^{\circ}{\text{Brix}}_{0}}$$

where °Brix_*t*_: concentration of the feed at time “*t*”, and Brix_0_: initial concentration.

#### 2.2.1 RO Membrane Cleaning

After every run, a clean‐in‐place (CIP) procedure was performed, consisting of 10 min rinse with RO water, followed by an alkaline wash with 0.1% sodium hydroxide and 0.025% sodium dodecyl sulfate, at 35°C, for 10 min. The membrane was then rinsed for 10 min with RO water, followed by 35 min enzymatic cleaning with 0.016% NaOH and 0.4% Hydryzime 399 (Hydrite Chemical Co., Brookfield, WI), at pH 10 and 35°C. This was followed by another 10 min rinse with RO water, 15 min alkaline wash as described above, and a final RO water rinse. The membrane water flux was measured before each run and after each cleaning. Cleaning was considered effective if water flux recovery was > 95%.

### 2.3 FO Processing

TCJ reconstituted to 10 °Brix was concentrated using a FO unit (Ederna, France) equipped with a cellulose triacetate membrane of 63 mm outside diameter, 530 mm length and 0.54 m^2^ filtration area. Tripotassium citrate was used as osmotic agent (OA) based on its performance (Beldie et al. 2025) and because citrate is naturally found in beet juice (Vasconcellos et al. 2016). The OA had 48.5 °Brix, and an osmotic pressure of 35,000 kPa.

The juice was pumped through a heat exchanger, at an initial feed pressure of 120 kPa, and then through the FO membrane, in contact with the active layer of the membrane; the retentate was recirculated. The OA was pumped through a heat exchanger, at an average flow rate (FR) of 6.5 ± 0.6 kg/h, and then through the FO membrane, in contact with the membrane's support layer side. The feed and OA were maintained at 25°C. The diluted OA was continuously collected and measured gravimetrically. The process was stopped when the membrane's operational limit was reached. The FO runs for TCJ were performed in triplicate. For the validation FO runs, single strength NFC juice (8.4 °Brix) was concentrated as described above; the FR of the OA for this run was < 4.2 L/m^2^h, due to equipment limitations.

The FO permeate flux (L/m^2^h) was calculated as follows:

(3) $$J=\frac{\left(m_{\mathrm{DOA}2}-m_{\mathrm{DOA}1}\right)-\left(m_{\mathrm{OA}1-}m_{\mathrm{OA}2}\right)}{A\times t\times \rho }$$

where *m*_*D*OA1_ and *m*_*D*OA2_: masses (kg) of the diluted OA at times 1 and 2, *m*_OA1_ and *m*_OA2_: masses of the OA spent at times 1 and 2, *A*: filtration area (m^2^), *t*: time (h), *ρ*: permeate density (1 kg/L).

The feed concentration was measured every 15 min and the concentration factor calculated (Equation 2). Concentrate samples were collected in amber glass bottles and kept at 4°C ± 1°C until analyzed the following day.

FO membrane cleaning included a rinse with DI water on the feed side at 35°C, followed by a DI water rinse of the OA side, until OA concentration reached 0 °Brix. An alkaline enzymatic wash with 0.25% KHCO_3_ and 0.075% Ultrasil 67 (Ecolab, Saint Paul, MN) was then performed on the feed side for 35 min at 35°C. pH was monitored and adjusted to 7.8–8.0 with KHCO_3_. This was followed by three DI water rinses and a subsequent acid wash using 0.5% Ultrasil MP (Ecolab, Saint Paul, MN) and 0.2% sodium citrate, for 30 min at 35°C. pH was adjusted to 3.0–3.3 during cleaning by adding Ultrasil MP. This was followed by a triple DI water rinse. To prevent microbial growth, disinfection was performed on the feed and OA side for 15 min at 30°C, using 0.5% Oxonia active (Ecolab, Saint Paul, MN), followed by three 2 min DI water rinses.

### 2.4 RO–FO Combination Process

RO was conducted as described above, and FO was performed with 14 L of TCJ of 17 °Brix, or NFC juice of 11.5 °Brix, corresponding to the plateau concentrations reached by RO for the two juices. Due to membrane limitations, the average FR of the OA for this set of experiments was 3.4 kg/h.

### 2.5 Physicochemical Analysis

Juice concentration was determined as total soluble solids (TSS, in °Brix), using a digital refractometer (Sper Scientific 300,053‐Scottsdale, AZ). pH was measured at 25°C with an Orion Versa Star Pro Meter (Thermo Fisher Scientific, Chelmsford, MA) and water activity (*a*_*w*_) with an AquaLab Series 3 hygrometer (AquaLab, San Francisco, CA). Turbidity was determined using a LaMotte 2020wi Turbidimeter (LaMotte Company, Chestertown, MD) and was 8.4 °Brix for both NFC and TCJ.

Juice color (CIE L*a*b* values) was analyzed using a UltraScan VIS colorimeter (HunterLab, Reston, Virginia, USA); the color difference (Δ*E*) between the initial juice and the membrane processed juice was determined.

Titratable acidity (TA, in g/L of anhydrous citric acid) was determined using an EasyPlus Automated Titrator (Mettler Toledo LLC, Columbus, OH) with a titration endpoint pH 8.1, and calculated as follows:

(4) $$\mathrm{TA}=\frac{V_{\text{NaOH}}\times 0.1\times 64}{V_{s}}$$

where *V*_NaOH_: volume of added NaOH (mL), 0.1: NaOH molarity, 64: anhydrous citric acid equivalence factor, *V*_*s*_: sample volume (mL).

#### 2.5.1 Quantification of Betalains

Betacyanins (BC) and betaxanthins (BX) were quantified using a modification of the method by Nilsson (1970). Samples were diluted with a pH 6.5 phosphate buffer, and absorbance was measured with a UV–Vis GENESYS 20 spectrophotometer (Thermo Scientific, Waltham, MA) at 476, 538, and 600 nm. Absorbance values of betaxanthins (ABX), betacyanins (ABC), and impurities (AZ) were determined as follows:

(5) $$\mathrm{ABX}=1.095\times \left(A538-A600\right)$$

(6) $$\mathrm{ABC}=A476-A538-\frac{\mathrm{ABX}}{3.1}$$

(7) AZ=A538−ABX

The pigment molar concentrations (*C*) were calculated as follows:

(8) $$C=\frac{A\times \mathrm{DF}\;}{\varepsilon \times l}$$

where *A*: absorbance of each component, DF: dilution factor, *Ɛ*: molar extinction coefficient (1120 for betacyanins, 339 for betaxanthins), *l*: path length (1 cm).

The determined BC was directly correlated to the betanin concentration, which constitutes ~95% of BC (Nilsson 1970).

Particle size and zeta potential analyses were performed at 22°C using a Brookhaven 90Plus particle size analyzer (Brookhaven Instruments, Holtsville, NY). Samples were diluted to achieve a signal strength of 300–500 kcps. For particle size analysis, measurements consisted of five consecutive runs of 30 s each, at a fixed angle of 90°, with a refractive index of 1.33, and a dust‐filter cut‐off of 30.

#### 2.5.2 Reverse Solute Flux (RSF)

Potassium and citrate in FO concentrated NFC juice samples were quantified by ion chromatography (IC) for the initial product sample, an intermediate sample collected after 195 min of FO, and the final concentrated sample, collected at 360 min. The reverse solute flux (RSF) was determined according to (Beldie et al. 2025):

(9) $$\mathrm{RSF}=\frac{C_{s}}{A\times t}$$

where *A*: filtration area (m^2^), *t*: processing time (h).

The concentration of OA solutes in the feed (*C*_*s*_, mg/L) was calculated as follows:

(10) $$C_{s}=\frac{Q_{s}}{V_{i}-J_{\mathrm{avg}}\times A\times t}$$

where *Q*_*s*_: concentration of each ion (mg/g), *V*_*i*_: initial feed volume (L), *J*_avg_: average permeate flux (L/m^2^h). Feed density was considered 1 kg/L.

### 2.6 Statistical Analysis

Data was analyzed by one‐way ANOVA with Tukey's HSD post hoc test to determine significance at a 95% confidence level, using RStudio software (2023).

## 3 Results and Discussion

### 3.1 Beetroot Juice Concentration by RO and FO

**FIGURE 1:**
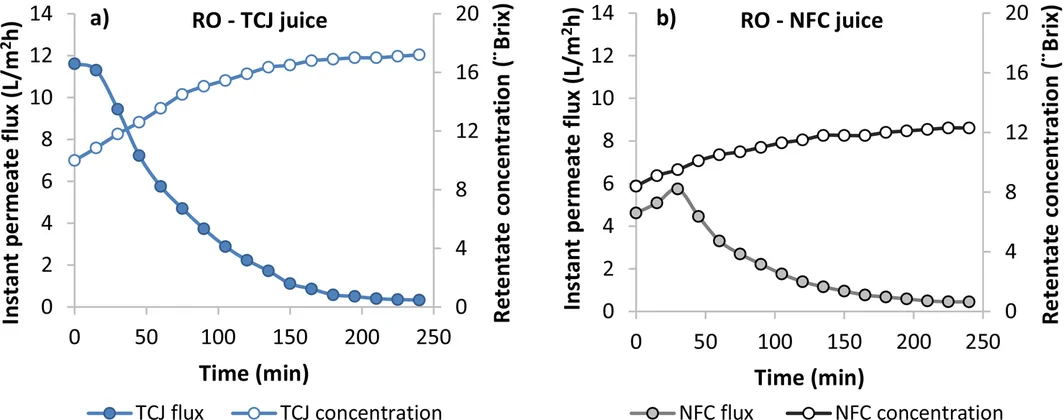
Examples of permeate (water) flux and concentration versus time curves for RO of (a) TCJ and (b) NFC juice.

Water flux during RO decreased with time while concentration increased until a plateau was reached (Figure 1). For TCJ, RO started at 10 °Brix and after 240 min an average of 17.3 °Brix ± 0.4 °Brix across three RO runs was reached (Figure 1a), corresponding to CF = 1.7. For the NFC juice (8.4 °Brix), 12.3 °Brix was reached after 240 min of RO (Figure 1b), corresponding to CF = 1.5.

**FIGURE 2:**
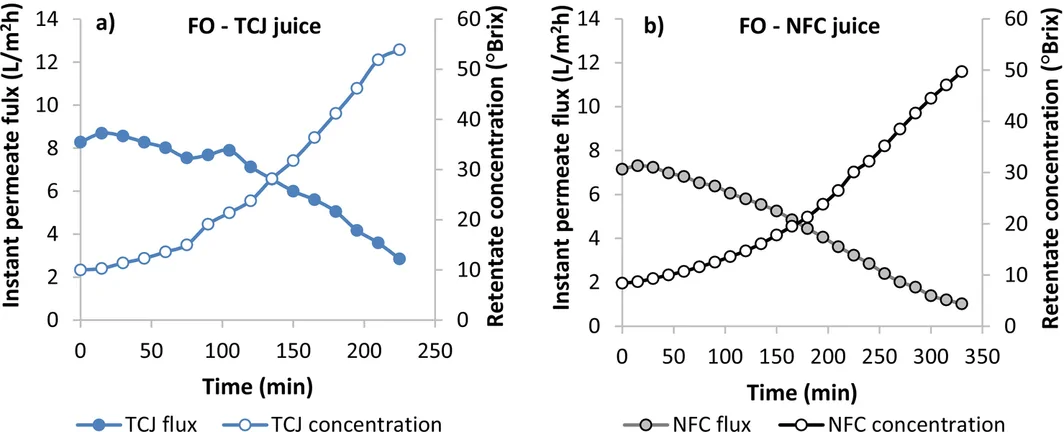
Examples of permeate flux and concentration versus time curves for FO concentration of (a) TCJ and (b) NFC, starting at single strength.

The average highest permeate flux for RO of TCJ was 12.5 ± 0.8 L/m^2^h, whereas for the NFC juice the highest permeate flux was 5.9 L/m^2^h. As a note, the observed flux increase at the beginning of RO of NFC juice was due to a low starting feed temperature (14°C) for this run; the temperature stabilized at 22°C after 20 min, when the flux reached its highest value. The permeate flux during RO of NFC was considerably lower than for TCJ, which was attributed to differences in composition and prior processing between the two juices. The observed RO permeate fluxes were lower than for orange juice (Jesus et al. 2007), peach, pear, apple, and mandarin juice (Echavarría et al. 2012), or acid whey (Menchik and Moraru I 2019), raw but similar to watermelon juice (Quoc et al. 2022), likely due to differences in initial soluble solids of the feed and the TMP used (Figure 2).

In FO, TCJ was concentrated from 10 °Brix to 50.9 ± 2.9 °Brix (Figure 2a), or CF = 5.1, while NFC was concentrated from 8.4 °Brix to 49.7 °Brix (Figure 2b), or CF = 5.9. The longer duration of the NFC run was due to the lower starting concentration than TCJ. FO had to be stopped before a concentration plateau was reached due to system pressure limitations, which suggests that a higher final concentration is achievable; Trishitman et al. (2021) reached 61.4 °Brix during FO concentration of clarified beetroot juice.

FO flux decreased from an initial value of 8.1 ± 0.2 L/m^2^h to a final flux of 1.9 ± 0.1 2 L/m^2^h for TCJ, and from 7.2 L/m^2^h to 1.0 L/m^2^h for NFC. Trishitman et al. (2021) reported a higher initial FO flux (12.2 L/m^2^h), but a similar final flux (1.29 L m^2^h) for clarified beetroot juice.

**FIGURE 3:**
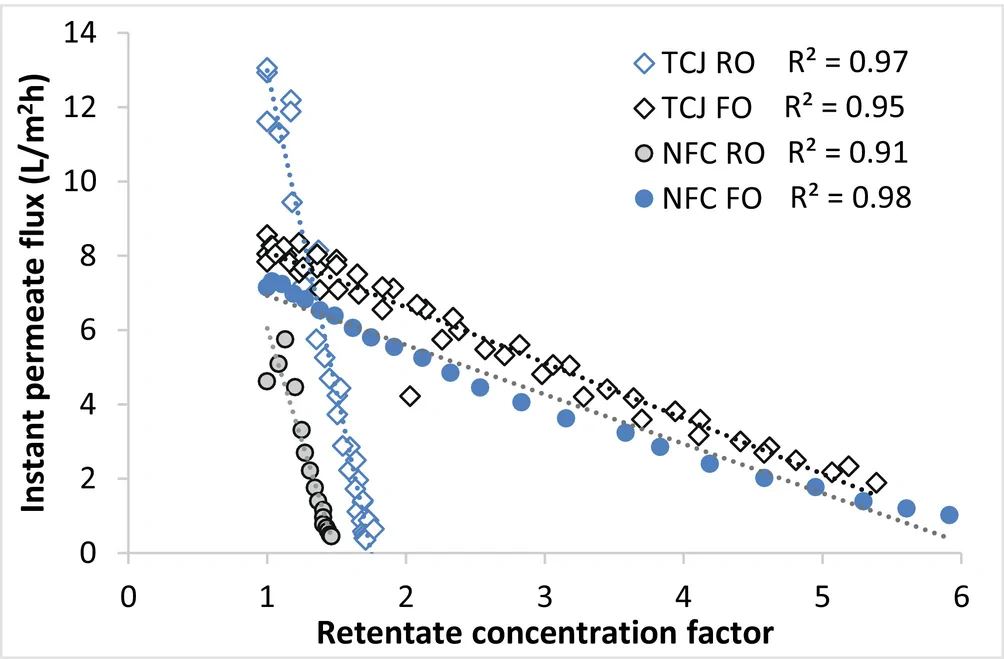
Permeate (water) flux versus concentration factor for the RO and FO of TCJ juices (data points for all three runs), and for the NFC (one validation run).

FO fluxes were smaller than in RO, although the difference was smaller than for other feeds (Menchik and Moraru I 2019). A more appropriate comparison between the two processes is offered by the flux versus CF (Figure 3), which shows a much faster flux decline in RO compared to FO. The point where RO flux dropped below the FO flux for TCJ was at ~13 °Brix, or CF ~1.3. The flux drop (J/J0) in RO exceeded 50% after 40 min for TCJ and after 70 min for NFC, while in FO, J/J0 reached 50% after 190 min for TCJ and after 210 min for NFC. This is a clear indication of more severe fouling in RO.

**FIGURE 4:**
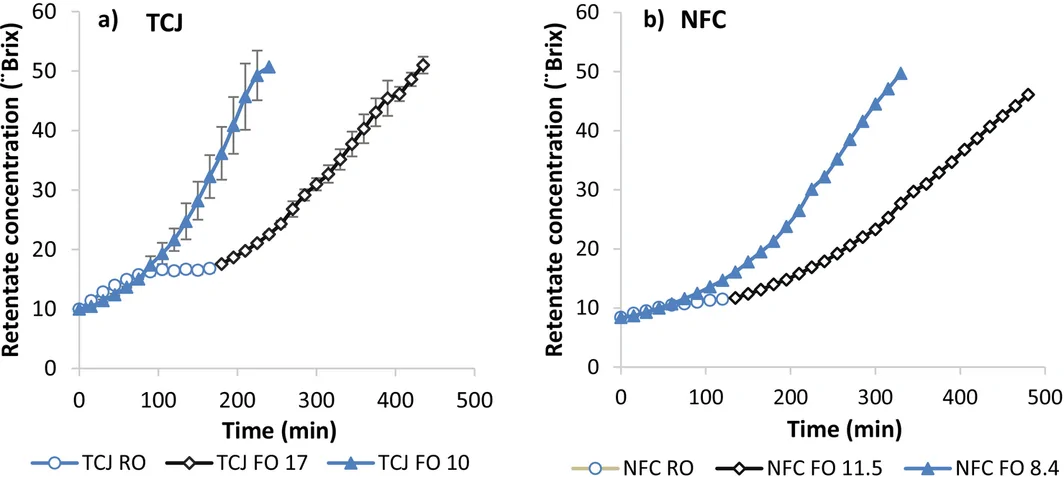
Retentate concentration versus time for the RO–FO combined process and individual FO concentration for (a) TCJ (*n* = 3) and (b) NFC (single validation run). For the FO runs, the number on the label indicates the starting concentration of the feed, in °Brix.

**FIGURE 5:**
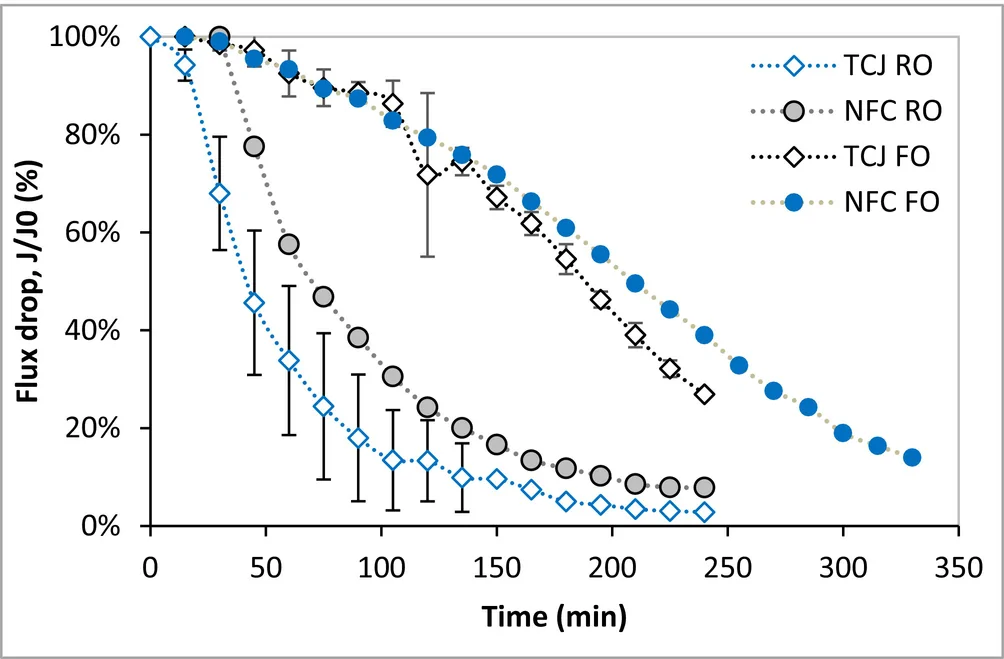
Flux drop (*J*/*J0*, flux as a percentage of the initial flux) versus time for the RO and FO of TCJ (error bars represent 1 SD, *n* = 3) and NFC (single validation run).

Previously, (Menchik and Moraru I 2019) demonstrated that coupling a preliminary RO step and a subsequent FO step would take advantage of RO's higher fluxes at low feed concentration and FO's low fouling propensity at high concentrations. This RO–FO combination treatment was tested for both TCJ and NFC. For TCJ, juice was RO concentrated to 17 °Brix, followed by FO up to 50.2 ± 1.7 °Brix (Figure 4a); the average duration (*n* = 3) of the combination process was 435 min, with 255 min for the FO step. By comparison, the FO‐only concentration of TCJ reached a similar concentration (50.7 °Brix) in only 240 min. For NFC juice, RO achieved 11.5 °Brix, and the subsequent FO step reached 46.1°Brix in a total of 480 min (345 min for the FO step). By FO alone, NFC concentration reached 49.7 °Brix, but in only 330 min (Figure 4b). Like in the individual runs, FO in the RO–FO combination had to be stopped before the concentration reached a plateau due to system pressure limitations (Figure 5).

For the FO step in the combination process, the initial and final permeate fluxes were 7.1 ± 0.6 and 6.3 L/m^2^h for the TCJ and NFC runs, respectively; the final permeate fluxes were 1.9 ± 0.2 and 1.1 L/m^2^h for TCJ and NFC, respectively, similar to fluxes achieved for FO‐only. Given the longer duration of the RO–FO combination compared to FO‐only, it appears that for beetroot juice the combination process does not have benefits. The main reason for this is suspected to be the significant fouling that occurred during RO.

### 3.2 An Indirect Assessment of Membrane Fouling During RO

While a thorough investigation of fouling was outside the scope of this work, the drop in flux clearly indicated the occurrence of membrane fouling. Fouling in FO is more complex and not easy to interpret based on flux data alone. However, in case of RO, flux data can be used to make inferences regarding the type of fouling based on the membrane fouling models proposed by (Hermia 1982): (i) complete pore blocking, (ii) standard pore blocking, (iii) cake filtration, and (iv) intermediate pore blocking. The fit with one of these scenarios can be determined based on the linear regression analysis of a transformation of the permeate flux (J) versus time: (ln(1/J)) versus time for complete pore blocking, (1/J^−0.5^) versus time for standard pore blocking, (1/J) versus time for intermediate pore blocking, and (1/J^2^) versus time for cake formation (Quoc et al. 2022; Garcia‐Castello et al. 2011; Wang and Tarabara 2008; Hermia 1982).

**FIGURE 6:**
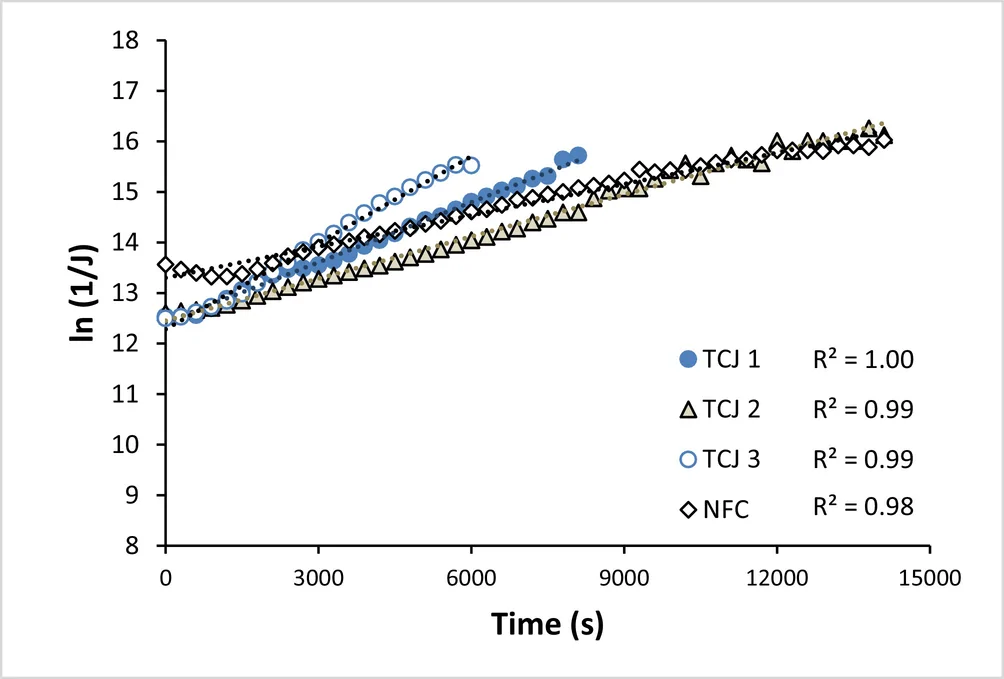
Application of the blocking laws of filtration for RO, according to Hermia's models (Hermia 1982). Linear regression was applied for the logarithmic transformation of permeate flux (ln(1/*J*)) versus time for the concentration of TCJ by RO and FO, and the concentration of NFC by RO and early stage FO. For late stage FO, the linear regression was applied for (1/*J*2) versus time.

In the current work, the best fit for RO (R^2^ between 0.98–1.00), for both TCJ and NFC, was obtained for ln(1/J) versus time (Figure 6a). This is consistent with reports for RO of other juices (Wang and Tarabara 2008; Garcia‐Castello et al. 2011; Quoc et al. 2022) and suggests that complete pore blocking is the dominant membrane fouling mechanism. While osmotic processes use dense membranes without clearly defined pores, a good fit by the complete or intermediate blocking models for dense membranes was explained before by the presence of surface defects that function as “pores” during initial stages of filtration (Garcia‐Castello et al. 2011; Wang and Tarabara 2008). For FO, although some pore blocking might be encountered on the feed side of the membrane, the process is affected by more complex phenomena than RO, and therefore the fouling models discussed above are not applicable for FO.

#### 3.2.1 Effect of Juice on Membrane Fouling

Since permeate fluxes were lower for NFC compared to TCJ for both processes, even if the initial concentration was lower for NFC (8.4 °Brix) than TCJ (10 °Brix), the impact of juice properties on membrane fouling was assessed.

**FIGURE 7:**
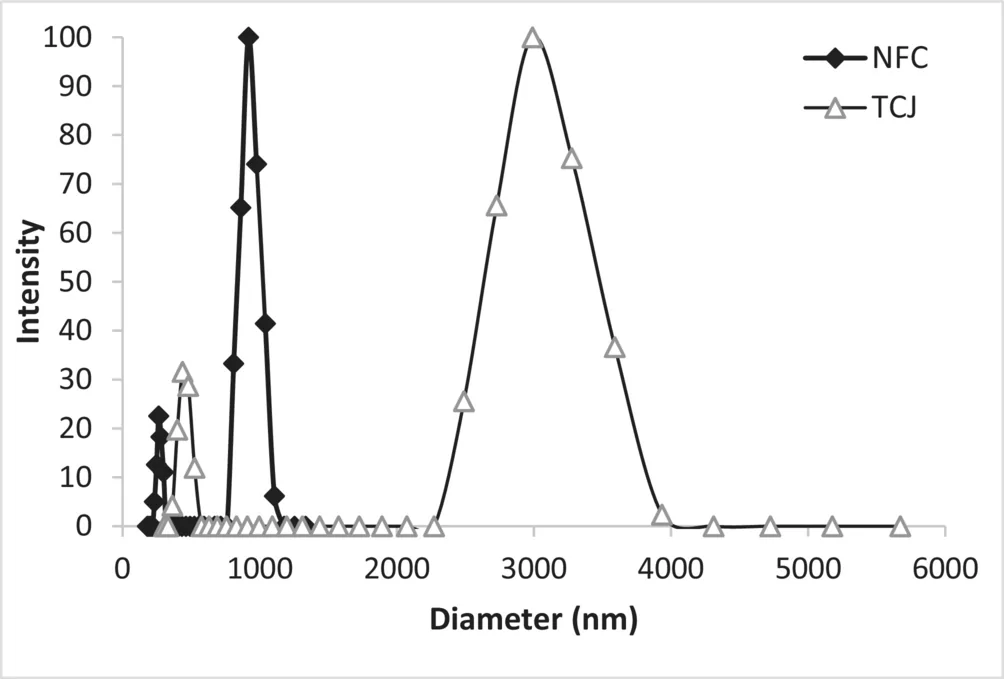
Particle size distribution for NFC and TCJ, prior to membrane concentration.

ζ potential values for NFC and TCJ were−22.87 ± 1.86 mV and−23.39 ± 1.15 mV, respectively, and were not significantly different from each other (*p* > 0.05). Thus, neither juice had a higher propensity for interacting electrostatically with the membrane surface. However, the particle size distributions for the two juices showed differences. Both samples exhibited bimodal distributions (Figure 7): in NFC, the particle size groups were centered around 250 nm and 1000 nm, respectively, while for TCJ they were centered around 500 nm and 3000 nm, respectively. The larger population in TCJ likely represents protein‐polyphenols complexes formed during thermal processing (Zhu et al. 2021).

NFC had a turbidity of 168 ± 1 NFU, compared to 142 NFU for TCJ, indicating a greater particle density for NFC. This, combined with the smaller particle sizes, likely led to more pronounced blocking of membrane surface by the NFC, explaining the lower fluxes obtained for this juice for both RO and FO. While these explanations are reasonable, and in line with general knowledge about membrane fouling, these interferences will need to be tested in a future study focused on fouling mechanisms during membrane concentration of red beet juice.

### 3.3 Physicochemical Properties of the RO and FO Concentrated Beetroot Juice

The physicochemical properties for TCJ and NFC juices, before and after membrane concentration and reconstitution to single strength, are shown in (Table 1). For TCJ, the only significant differences (*p* < 0.05) induced by membrane processing were observed for pH and TA for the FO juice. FO led to an increase in pH, from 4.35 to 4.69, and a 20% decrease in TA. The NFC juice concentrated by FO showed a pH increase from 3.83 to 4.90, and a 60% reduction in TA. (Chu et al. 2022) also observed a pH increase in blueberry juice concentrated by FO when using potassium sorbate and sodium benzoate as osmotic agents, which they attributed to reverse solute flux (RSF). This is likely the reason for the changes in pH and acidity in the current study, as will be discussed later.

**TABLE 1:** Physicochemical parameters of TCJ and NFC before processing, and after membrane concentration and reconstitution to single strength. Data represents averages ±1 stdev for three concentration runs and three technical replicates per run for TCJ, and three technical replicates for the one validation run for NFC.

Sample	TSS, °Brix	pH	TA, g/L	a_w_	L*	a*	b*	ΔE	Betacyanin*, BC (mg/L)	Betaxanthin*, BX (mg/L)
TCJ, before RO	10.0	4.40 ± 0.03^b^	3.46 ± 0.04^a^	1.00 ± 0.00^a^	17.09 ± 0.33^a^	10.36 ± 1.48^a^	4.51 ± 0.21^a^		654.49 ± 25.43^a^	239.07 ± 18.13^a^
TCJ, RO & reconst.	10.0	4.40 ± 0.03^b^	3.43 ± 0.06^a^	1.00 ± 0.00^a^	17.16 ± 0.09^a^	10.76 ± 1.16^a^	4.62 ± 0.27^a^	0.42	653.18 ± 21.28^a^	243.78 ± 3.98^a^
TCJ, before FO	10.0	4.35 ± 0.01^b^	3.41 ± 0.29^a^	1.00 ± 0.00^a^	16.80 ± 0.50^a^	9.13 ± 1.55^a^	3.82 ± 1.19^a^		642.35 ± 39.59^a^	233.72 ± 17.95^a^
TCJ, FO & reconst.	10.0	4.69 ± 0.05^a^	2.79 ± 0.18^b^	1.00 ± 0.00^a^	16.66 ± 0.64^a^	9.89 ± 1.73^a^	3.99 ± 1.13^a^	0.79	615.45 ± 29.70^a^	241.41 ± 11.02^a^
NFC, before RO	8.4	3.86 ± 0.01^C^	7.22 ± 0.23^A^	1.00 ± 0.00^AB^	16.40 ± 0.04^C^	24.50 ± 0.53^B^	10.27 ± 0.34^bc^		153.04	76.97
NFC, RO& reconst.	8.4	3.88 ± 0.00^B^	6.76 ± 0.29^A^	0.99 ± 0.00^B^	17.53 ± 0.27^AB^	27.45 ± 0.54^A^	12.18 ± 0.33^A^	3.69	147.63	82.23
NFC, before FO	8.4	3.83 ± 0.00^D^	7.13 ± 0.48^A^	0.99 ± 0.00^B^	17.70 ± 0.48^A^	26.05 ± 1.45^AB^	11.49 ± 1.08^AB^		147.63	78.88
NFC, FO & reconst.	8.4	4.90 ± 0.01^A^	2.58 ± 0.68^B^	1.00 ± 0.00^A^	16.76 ± 0.45^bc^	18.39 ± 0.42^C^	8.82 ± 0.26^C^	8.17	140.25	94.23

*Note:* Values in each column with different lowercase letters are significantly different (*p* < 0.05) for TCJ. Values in each column with different capital letters are significantly different (*p* < 0.05) for NFC.

* Only one replicate is available for betalain (BC and BX) concentration in NFC.

In terms of color, the initial single strength TCJ had lower a* (weaker red hue) and b* values (weaker yellow hue) than NFC, even if TCJ had higher total solids (10 °Brix) compared to NFC (8.4 °Brix). Membrane concentration, particularly FO, resulted in color changes for NFC, which exhibited reductions in a* and b*, indicating a shift toward green and blue chromaticity. Similarly, Trishitman et al. (2021) reported a decrease in color parameters for beetroot juice after FO concentration. Color changes were insignificant for TCJ, which already underwent color changes during the prior thermal processing.

For NFC, the initial betalain content was smaller than for TCJ, likely due to differences in both processing and red beet cultivar. For TCJ, no significant changes in betalain content (BC, BX) were found after either RO or FO concentration, which is similar to observations by (Mereddy et al. 2017) for RO and Trishitman et al. (2021) for FO concentration of beetroot juice. For the NFC validation study (one replicate only), a 4%–5% reduction in betalain content was observed after RO and FO concentration.

For the RO–FO combination, TCJ composition did not change during RO, but after FO concentration of the 17 °Brix RO pre‐concentrate, the juice reconstituted to single strength showed a significant difference (*p* > 0.05) in both pH (increase) and TA (decrease) (Table 2). These differences were more pronounced in the NFC samples, which underwent a 20% increase in pH and a 60% reduction in TA. No significant differences in the color parameters occurred for TCJ, while for NFC a significant shift toward a green hue was observed. The betalain concentration did not change for either type of juice, but FO processing resulted in a slight reduction in betacyanin content for NFC, possibly due to air exposure during the lengthy FO processing in the open, small scale FO unit; this can likely be avoided in a closed FO system.

**TABLE 2:** Physicochemical parameters of preconcentrated TCJ and NFC before membrane processing and after concentration and reconstitution to single strength. Data represents averages ±1 SD for three concentration runs and three technical replicates per run for TCJ, and three technical replicates for the one validation run for NFC.

Sample	TSS, °Brix	pH	TA, g/L	a_w_	L*	a*	b*	ΔE	Betacyanin*, BC (mg/L)	Betaxanthin*, BX (mg/L)
TCJ, before FO	17.0	4.43 ± 0.06^b^	4.92 ± 0.33^a^	0.99 ± 0.00^a^	16.59 ± 0.77^a^	4.46 ± 1.36^a^	3.47 ± 0.20^a^		1131.66 ± 74.32^a^	422.24 ± 27.18^a^
TCJ, FO & reconst.	17.0	4.73 ± 0.06^a^	4.24 ± 0.49^a^	0.99 ± 0.00^a^	16.94 ± 0.95^a^	3.36 ± 0.38^a^	3.12 ± 0.26^a^	1.21	1105.91 ± 32.20^a^	435.34 ± 16.07^a^
NFC, before FO	11.5	4.01 ± 0.01^B^	7.56 ± 0.53^A^	0.99 ± 0.00^B^	17.33 ± 0.18^A^	23.23 ± 0.69^A^	9.90 ± 0.17^A^		186.01	123.75
NFC, FO & reconst.	11.5	4.95 ± 0.01^A^	2.61 ± 0.19^B^	0.99 ± 0.00^A^	16.62 ± 0.06^B^	17.39 ± 0.66^B^	8.11 ± 0.40^B^	6.15	177.16	134.25

*Note:* Values in each column with different lowercase letters are significantly different (*p* < 0.05) for TCJ. Values in each column with different capital letters are significantly different (*p* < 0.05) for NFC.

* Only one replicate is available for betalain (BC and BX) concentration in NFC.

#### 3.3.1 Reverse Solute Flux for FO Concentration

To check if migration of solutes from the OA into the juice occurred, potassium and citrate were quantified for NFC processed by FO in the combination process, at different stages: before FO (NFC_FO initial_, which is juice preconcentrated by RO to 11.5 °Brix), an intermediate sample collected after 195 min (NFC_FO itermed_, 25.3 °Brix), and a final sample, after 360 min of FO (NFC_FO final_, 46.1 °Brix). The change in concentration of potassium and citrate reverse solute flux, or RSF, was calculated according to (Equation 9).

As shown in (Table 3), citrate concentration increased in juice as FO progressed; the average RSF for citrate over 360 min of FO was 12 mg/m^2^h. The increase in citrate concentration was very small and amounted to a 3.7% increase by the end of FO. This slight increase in citrate concentration falls within the range of natural variability in citrate concentration for beetroot juice. However, this small increase in concentration in negatively charged citrate led to the neutralization of dissociated protons in juice, leading to the observed pH increase.

**TABLE 3:** Concentration and reverse solute flux (RSF) for citrate and potassium in NFC concentrated by FO.

Sample	Soluble solids (°Brix)	Citrate concentration (μg/g)	Citrate added by FO*, (μg/g)	Citrate RSF (mg/m^2^h)	Potassium concentration (μg/g)	Potassium added by FO* (μg/g)	Potassium RSF (mg/m^2^h)
NFC, FO initial	11.5	140 ± 6	—	—	5661 ± 431	—	
NFC, FO intermed.	25.3	312 ± 6	4 (1.3% increase)	11.16	13,904 ± 226	1449 (11.6% increase)	4044
NFC, FO final	46.1	582 ± 6	21 (3.7% increase)	20.32	21,573 ± 139	−1120 (4.9% decrease)	−1731

* The values for the concentration of solutes added by FO were calculated taking into consideration the FO juice concentration factor at the time of sampling.

Changes in potassium were more significant, which is to be expected considering the smaller molecular weight of potassium (39.10 g/mol) compared to citrate (189.10 g/mol). Potassium levels increased by 11.6% in the intermediate FO concentrate but surprisingly decreased by the end of FO (a 4.9% net decrease); this suggests migration of potassium from juice into the OA. This change may be related to a change in fouling mechanisms during FO, but this would require further study. From a practical perspective though, the net changes in potassium concentration are very small compared to the potassium concentration in juice.

Even if for both solutes the observed change in concentration in the juice falls within the natural variability of beetroot juice composition, a follow up sensory study is required to assess if these small changes in citrate and potassium concentration have an impact on the sensory characteristics of FO concentrated red beetroot juice.

### 3.4 Conclusions

This work demonstrates that FO is very effective for the nonthermal concentration of red beetroot juice. Although the study evidenced small reverse solute flux and pH shifts, FO was found to preserve the overall quality of beetroot juice and its thermolabile compounds, with only negligible effects observed in betacyanin and betaxanthin content. FO offers a considerably higher final product concentration compared to RO and a greater flux stability compared to both RO and the combined RO–FO process. These findings offer valuable insights regarding the promise that FO has as a gentle method for the concentration of red beetroot juice, both as a product with high nutritional value and an attractive source of natural red food color. Future studies will be required to assess how juice extracted from different beet cultivars impacts the concentration process and to elucidate mechanisms of fouling, which could offer better insights into how the process can be optimized for better outcomes.

## Author Contributions

**Eugenia Avellaneda:** data curation, formal analysis, methodology, visualization, writing – original draft, investigation. **Carmen I. Moraru:** conceptualization, supervision, project administration, writing – review and editing, funding acquisition, resources.

## Funding

This work was supported by the U.S. Department of Agriculture, NC1023.

## Conflicts of Interest

The authors declare no conflicts of interest.

## Data Availability

The data that support the findings of this study are available from the corresponding author upon reasonable request.
